# Combined Antitumor Effect of the Serine Protease Urokinase Inhibitor Upamostat and the Sphingosine Kinase 2 Inhibitor Opaganib on Cholangiocarcinoma Patient-Derived Xenografts

**DOI:** 10.3390/cancers16051050

**Published:** 2024-03-05

**Authors:** Faizal Z. Asumda, Nellie A. Campbell, Mohamed A. Hassan, Reza Fathi, Daniella F. Vasquez Rico, Melanie Kiem, Ethan V. Vang, Yo Han Kim, Xin Luo, Daniel R. O’Brien, Sarah A. Buhrow, Joel M. Reid, Michael J. Moore, Vered Katz Ben-Yair, Mark L. Levitt, Jennifer L. Leiting, Amro M. Abdelrahman, Xinli Zhu, Fabrice Lucien, Mark J. Truty, Lewis R. Roberts

**Affiliations:** 1 Departments of Pediatrics and Pathology, Medical College of Georgia-Augusta University Medical Center, Augusta, GA 30912, USA; fasumda@augusta.edu; 2 Division of Gastroenterology and Hepatology, Mayo Clinic College of Medicine and Science, Mayo Clinic Cancer Center, Rochester, MN 55905, USA; campbell.nellie@mayo.edu (N.A.C.); moore.michael.j@wustl.edu (M.J.M.); zhu.xinli@mayo.edu (X.Z.); 3 Southwest Medical Associates, Las Vegas, NV 89128, USA; 4 RedHill Biopharma, Ltd., 21 Ha’arba’a St., Tel Aviv 6473921, Israel; reza@redhillbio.com (R.F.); mark@redhillbio.com (M.L.L.); 5 Department of Microbiology & Immunology, University of Minnesota, Minneapolis, MN 55455, USA; vasqu170@umn.edu; 6 Study of Human Medicine, Paracelsus Medical University, Strubergasse 21, 5020 Salzburg, Austria; 7 Department of Urology, Mayo Clinic College of Medicine and Science, Rochester, MN 55905, USA; kim.yohan@mayo.edu (Y.H.K.); lucien-matteoni.fabrice@mayo.edu (F.L.); 8 Hepatic Surgery Center and Hubei Key Laboratory of Hepato-Pancreatic-Biliary Diseases, Tongji Hospital, Tongji Medical College, Huazhong University of Science and Technology, Wuhan 430074, China; 9 Department of Quantitative Health Sciences, Mayo Clinic College of Medicine and Science, Rochester, MN 55905, USA; obrien.daniel@mayo.edu; 10 Department of Oncology and Department of Molecular Pharmacology and Experimental Therapeutics, Mayo Clinic College of Medicine and Science, Rochester, MN 55905, USA; buhrow.sarah@mayo.edu (S.A.B.); reid@mayo.edu (J.M.R.); 11 Division of Subspecialty General Surgery, Department of Surgery, Mayo Clinic College of Medicine and Science, Rochester, MN 55905, USA; leiting.jennifer@mayo.edu; 12 Division of Hepatobiliary and Pancreatic Surgery, Department of Surgery, Mayo Clinic College of Medicine and Science, Rochester, MN 55905, USA; abdelrahman.amro@mayo.edu (A.M.A.); truty.mark@mayo.edu (M.J.T.); 13 Department of Radiation Oncology, The First Affiliated Hospital, Zhejiang University School of Medicine, Hangzhou 310030, China

**Keywords:** cholangiocellular carcinoma, upamostat, opaganib, WX-UK1, serine protease, sphingosine kinase, patient-derived xenograft (PDX)

## Abstract

**Simple Summary:**

Cholangiocarcinoma (CCA) accounts for approximately 15% of primary liver cancers. CCA has a poor prognosis and, thus, more effective systemic treatments are needed. We tested opaganib and upamostat, drugs that target sphingosine kinase 2 and multiple serine proteases, primarily trypsins, which are highly expressed in CCA tumors. This study demonstrates the results of inhibiting these novel targets with these drugs individually and in combination in a patient-derived CCA xenograft mouse model.

**Abstract:**

Upamostat is an orally available small-molecule serine protease inhibitor that is a highly potent inhibitor of trypsin 1, trypsin 2, trypsin 3 (PRSS1/2/3), and the urokinase-type plasminogen activator (uPA). These enzymes are expressed in many cancers, especially during tissue remodeling and subsequent tumor cell invasion. Opaganib (ABC294640), a novel, orally available small molecule is a selective inhibitor of the phosphorylation of sphingosine to sphingosine-1-phosphate (S-1-P) by sphingosine kinase 2 (SPHK2). Both sphingosine kinase 1 (SPHK1) and SPHK2 are known to regulate the proliferation-inducing compound S-1-P. However, SPHK2 is more critical in cancer pathogenesis. The goal of this project was to investigate the potential antitumor effects of upamostat and opaganib, individually and in combination, on cholangiocarcinoma (CCA) xenografts in nude mice. PAX165, a patient-derived xenograft (PDX) from a surgically resected CCA, expresses substantial levels of SPHK2, PRSS1, PRSS2, and PRSS3. Four groups of 18 mice each were treated with upamostat, opaganib, both, or vehicle. Mouse weights and PAX165 tumor volumes were measured. Tumor volumes in the upamostat, opaganib, and upamostat plus opaganib groups were significantly decreased compared to the control group.

## 1. Introduction

Cholangiocarcinoma (CCA) is an epithelial tumor arising in the intrahepatic (iCCA), perihilar (pCCA), or distal biliary tree (dCCA). It is the second most common type of hepatic malignancy, accounting for 15% of all primary liver cancers and 3% of gastrointestinal cancers [1]. Due to the rising incidence of CCA in industrial countries and limited treatment options, CCA is of increasing public health importance. CCAs are asymptomatic at early stages, highly aggressive, and usually diagnosed at an advanced stage [2,3]. Curative treatment options, including surgical resection, are indicated for early-stage CCA. For unresectable tumors, palliative systemic treatment consisting of gemcitabine, platinum-based chemotherapy, and immune checkpoint inhibitor therapy is the current standard of care [3,4]. In about 10% to 15% of iCCA patients, FGFR2 fusions are identified as key oncogenic drivers [5]. Other clinically significant alterations include IDH1 mutations, mismatch repair deficiency, or NTRK mutations [5]. These patients, who constitute a minority of the CCA patient population, are candidates for targeted chemotherapy or immunotherapy, although neither therapeutic strategy is known to be curative [5]. Chemotherapy resistance as well as resistance to immunotherapy is frequent and existing therapeutic options are of limited effectiveness, thus exploring new targeted treatments is essential.

Upamostat (WX-671, Mesupron) is an orally available, small-molecule serine protease inhibitor that is a highly potent inhibitor of trypsin 1, trypsin 2, and trypsin 3 (PRSS1/2/3), as well as urokinase-type plasminogen activator (uPA) [6]. WX-UK1, the active metabolite of upamostat, is an inhibitor of S1 trypsin-like serine proteases and is used for in vitro studies [7,8]. Proteolytic enzymes like serine proteases are commonly overexpressed in solid tumors, including CCA, and mediate extracellular matrix (ECM) degradation surrounding the tumor. This is crucial in tissue remodeling, promoting cancer invasion and metastasis [9]. As a proteolytic serine enzyme complex, the plasminogen activation system (uPAS) is essential for tissue remodeling, cellular invasiveness, metastasis, ECM degradation, and tumor growth [10,11,12,13,14]. Blocking uPAS and plasmin formation leads to decreased growth and metastatic potential of tumor cells [15,16]. Multiple tumor models have demonstrated the in vivo anti-metastatic and antiproliferative activity of WX-UK1 and its prodrug upamostat [7].

Opaganib (ABC294640, Yeliva), a novel, orally available small molecule, is a first-in-class selective inhibitor of sphingosine kinase 2 (SPHK2). Sphingolipid-metabolizing enzymes control the dynamic balance of the cellular levels of important bioactive lipids, including the apoptotic compound ceramide and the proliferative compound sphingosine 1-phosphate (S1P), which is regulated by the sphingosine kinases SPHK1 and SPHK2, although SPHK2 appears to be more involved in cancer [17,18]. The SPHK2-specific inhibitor opaganib showed high efficacy in several preclinical cancer models and synergistic anticancer activity with chemotherapies or molecularly targeted therapies [19,20]. Anti-tumor activity of opaganib is mediated by multiple underlying mechanisms, including direct inhibition of cell proliferation [19], enhanced apoptosis of CCA cells through the upregulation of pro-apoptotic NOXA [21,22], and the induction of CCA cell autophagy [23].

Both upamostat and opaganib are in the clinical stage of development and are safe for human use [24,25,26]. We aimed to investigate the potential antitumor effect of upamostat and opaganib, individually and in combination, on CCA patient-derived xenografts (PDX) in nude mice using PAX165, a cholangiocarcinoma PDX that expresses substantial levels of SPHK2, PRSS1, PRSS2, and PRSS3.

## 2. Materials and Methods

### 2.1. In Vivo Treatment

The care and use of the animals for these studies were reviewed and approved by the Institutional Animal Care and Use Committee of the Mayo Clinic College of Medicine and Science.

#### 2.1.1. Mouse–Human F1/F2 PDX Creation/Tumor Engraftment

According to the protocol approved by the Institutional Review Board (IRB) and the Institutional Animal Care and Use Committee (IACUC) of the Mayo Clinic College of Medicine and Science, tumors were minced into approximately 1 × 2 mm, coated with Matrigel (Corning, Corning, NY, USA) and engrafted into subcutaneous flank pockets of NOD/SCID mice (primary engraftment) [27,28,29]. Mice were treated with a one-time single dose of rituximab (0.1 mg/mL, anti-CD20, Genentech, South San Francisco, CA, USA) via intraperitoneal injection to minimize the development of lymphomas. All generated tumors were evaluated for histomorphology using H&E, comparing PDX tumors with original patient tumor slides by a Mayo Clinic GI pathologist. Time to tumor formation (TTF) was defined as the days from implantation to the first confirmed palpable tumor growth (approximately 3–4 mm). Time to tumor treatment (TTT) was defined as the number of days from implantation to when the tumor reached a volume of approximately 90–180 mm^3^ [27,28,29].

#### 2.1.2. Opaganib and Upamostat Treatment of PDX Tumor-Bearing Mice

The CCA PDX tumor (PAX165), was selected from a panel of 19 CCA PDXs based on its significant expression of the known cellular targets of upamostat, and opaganib (SPHK2, PRSS1, PRSS2, and PRSS3). Mice were randomly divided into 4 groups of 18 mice each and treated by oral gavage with either 70 mg/kg of upamostat (RedHill Biopharma, Tel Aviv, Israel), 50 mg/kg of opaganib (RedHill Biopharma), a combination of both, or vehicle (phosphate buffer). Mice were treated once a day for 6 weeks and sacrificed by CO_2_ inhalation on the first day of the seventh week. Tumor volumes and body weights were measured three times a week using calipers and a balance. Tumor volume was calculated according to the formula (a × b^2^)/2, where a and b are the long and short tumor diameters, respectively. The mice were sacrificed by CO_2_ inhalation at 7 weeks after treatment initiation. Tumors were dissected from the mice and examined by H&E staining and immunohistochemistry. Additional tissues were collected for further pharmacokinetics (PK) analysis including 1.2 mL of whole blood (collected via cardiac puncture using heparinized syringes and processed for plasma), muscle, and liver tissue.

#### 2.1.3. Pharmacokinetic (PK) Analysis

Mouse plasma samples were extracted in methanol. Separation of upamostat and its active metabolite WX-UK1, and opaganib, was accomplished via liquid chromatography using an Acquity UPLC BEH C18 analytical column. Upamostat, WX-UK1, and opaganib were detected via mass spectrometry (see Appendix A for the detailed protocol).

#### 2.1.4. Ki67 Cell Proliferation Analysis

Consecutive cryosections (4 μm) of each tumor were fixed in acetone (10 min, RT) and incubated in H_2_O_2_ (10 min, RT, 0.03%) to block endogenous peroxidase activity. Proliferation rates were measured as the percentage of Ki67-positive cells in tumors. The presence of Ki67-positive tumor cells was analyzed using a confocal fluorescence laser scanning system.

#### 2.1.5. TUNEL Assay for Cell Death Detection

Tissues were fixed in 4% PFA (20 min, RT), washed in 1× PBS (30 min, RT), and incubated in permeabilization solution (2 min, 4 °C). Positive control slides were incubated in 10 uL of DNase + 70 uL buffer (10 min, RT). TUNEL RXN mixture (50 uL enzyme solution, 450 uL label solution) was added to each sample, incubated with DAPI (1:5000 in 1× PBS, 5 min, RT), and analyzed under a fluorescence microscope.

#### 2.1.6. Immunohistochemistry 

Formalin-fixed paraffin-embedded (FFPE) tumor tissues from each of the treated mice were placed on glass slides. The tissues were deparaffinized and hydrated through xylene and a graded alcohol series. This was followed by permeabilization with 0.1% Triton X-100 in PBS, antigen retrieval with 10 mM sodium citrate buffer, and quenching of endogenous peroxidase activity with 0.3% H_2_O_2_. 5% BSA was used to block the tissues for 1 h at room temperature. Primary antibodies against SPHK2 (antibody: D2V3G) and the serine proteases [trypsin 1 and 3 (PRSS1, PRSS3), and putative trypsin 6 (PRSS3P2)] (antibody Abcam 200997) were used at concentrations of 1:500 and 1:1000 respectively, incubating overnight. The EnVision+ Dual Link System-HRP kit (Dako) was used as a secondary antibody. DAB Substrate Kit, Peroxidase (Vector Laboratories) was used to precipitate, at the location of the HRP, which was later visualized using light microscopy at 20× and 40× magnification.

### 2.2. In Vitro Assay and Staining

#### 2.2.1. Cholangiocarcinoma Cell Line 

The established intrahepatic cholangiocarcinoma (iCCA) cell line HuCCT1 was cultured in RPMI1640 medium (Gibco) supplemented with 5% fetal bovine serum and 0.1% Primocin (Invitrogen, Waltham, MA, USA) at 37 °C in a 5% CO_2_ incubator.

#### 2.2.2. Cell Migration Assay 

The cell migration assay was performed using the IBIDI wound healing assay. Using the 2-well µ-Dish 35 mm from IBIDI, cholangiocarcinoma cell lines were plated for 24 h. The culture inserts in the µ-Dish were removed to create the wounds. The wounds were photographed with a phase-contrast microscope at 0 and 12 h. Cell migration was quantitated by measuring the width of each wound. The experiments were repeated 3 times.

#### 2.2.3. Cell Viability Assay 

To determine the effects of different treatments on cell survival, HuCCT1 cells were seeded into 96-well plates in triplicate at densities between 2000 and 5000 cells/well. The cells were treated with varying concentrations of upamostat and opaganib for 0, 6, 12, and 24 h. Cell viability was measured and averaged. Hoechst solution (BioLegend, San Diego, CA, USA) was used to identify cells regardless of viability, whilst Propidium Iodide Solution (BioLegend) was used to stain the dead cells.

### 2.3. Data and Statistical Analysis

Statistical analysis was performed using GraphPad Prism 9. A *p*-value of 0.05 was considered statistically significant. Data from the PK study was acquired and analyzed by Waters MassLynx v4.1 software.

## 3. Results

### 3.1. PAX165 Expresses Significant Levels of Sphingosine Kinase 2 and Serine Proteases 1, 2 and 3

**Figure 1:**
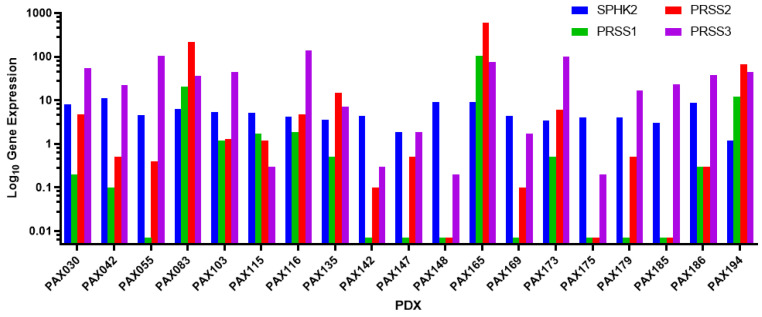
19 RNA sequencing was performed on 19 PDX lines established at the Mayo Clinic. Expressions of sphingosine kinase 2 (SPHK2), trypsin 1 (PRSS1), trypsin 2 (PRSS2), and trypsin 3 (PRSS3) were compared. All 19 PDXs showed expression of SPHK2 and trypsin 3; however, PAX165 showed substantial expression of all 4 proteins, making it ideal for the study.

RNA sequencing data from a panel of 19 CCA PDXs established at the Mayo Clinic was analyzed to identify the most suitable PDX that expressed all the target genes at high levels [30]. The target proteins were sphingosine kinase 2, trypsin 1, trypsin 2, and trypsin 3. The respective genes for these proteins were SPHK2, PRSS1, PRSS2, and PRSS3. PAX165 had the highest expression of these proteins when compared to the other xenografts, making it the best for the study (Figure 1). Of note, sphingosine kinase 2 (SPHK2) and trypsin 3 (PRSS3), which are the main targets of opaganib and upamostat, respectively, were expressed at high levels. 

### 3.2. Verification of Likeness between Original Tumor and Xenografts

Short tandem repeat (STR) analysis was performed to compare the characteristics of the second generation of the mouse xenograft (F2) and the xenografts implanted for the in vivo experiment (Table 1). This was done to confirm that the same tumor that was originally obtained from the patient was being used for this study.

To further validate genetic uniformity between the original tumor (F0) and the first-generation mouse xenograft (F1), H&E staining was performed. H&E staining revealed that the tumors were morphologically similar (Figure S1).

### 3.3. Opaganib and Upamostat Suppress CCA Tumor Growth in Mice

**Figure 2:**
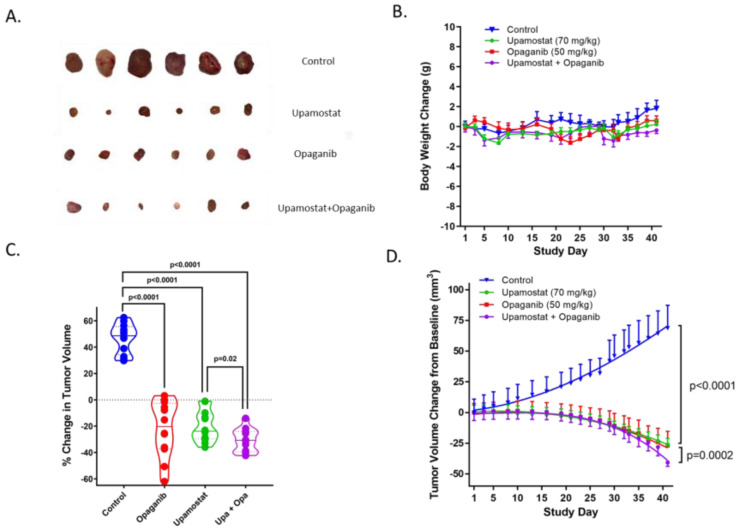
(**A**). Representative tumor nodules at the study endpoint are shown. (**B**). Body weights of the mice in the different groups were monitored, and no significant changes were seen within or between the groups. (**C**). At the study endpoint, tumor volumes of the upamostat, opaganib, and upamostat plus opaganib treated groups were significantly decreased compared to the control group. *p* < 0.0001 by *t*-test. The suppressive effect of upamostat plus opaganib on tumor growth was greater than that of upamostat alone, *p* < 0.02 by *t*-test. (**D**). Tumor volume changes from baseline were monitored. Compared to the control group, upamostat and opaganib each significantly reduced tumor growth in mice (*p* < 0.0001). When administered together, upamostat plus opaganib suppressed tumor growth to a greater degree than upamostat alone (*p* = 0.0002).

To assess the anti-tumor effects of opaganib and upamostat, tumor growth was measured in treated versus untreated mice. Upamostat and opaganib significantly suppressed tumor growth compared to the non-treated control group (*p* < 0.0001, Figure 2C,D). Combining both treatments resulted in greater growth inhibition, with a reduction in tumor volume, than upamostat alone or opaganib alone (*p* = 0.0002, Figure 2D). Tumor growth suppression mediated by opaganib and upamostat treatment was not accompanied by a decrease in body weight (Figure 2A,B).

### 3.4. Opaganib and Upamostat Modulate Drug Target Expression in CCA Tumors 

**Figure 3:**
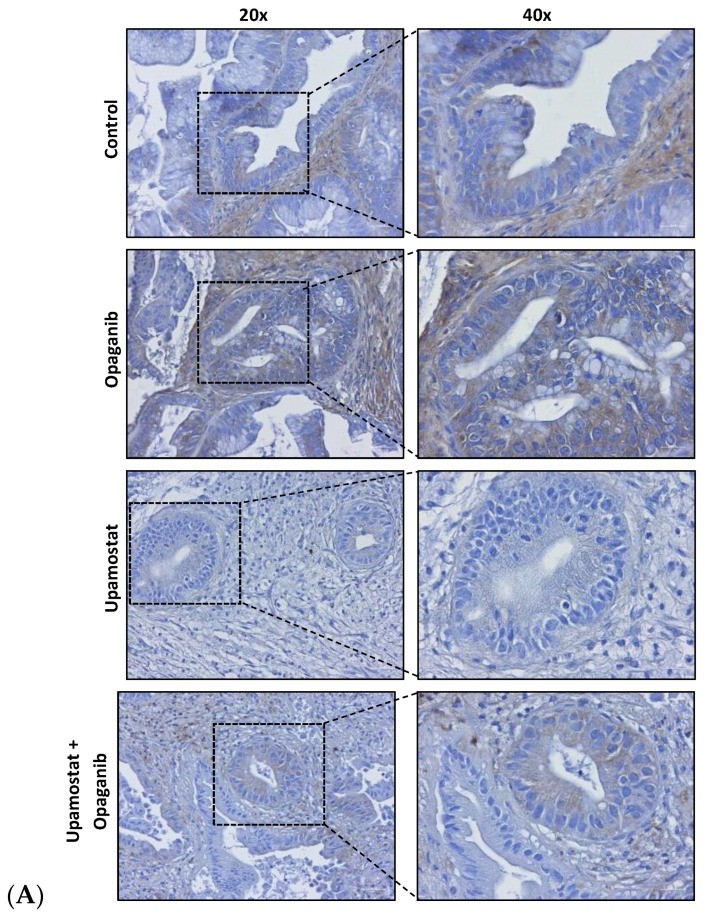

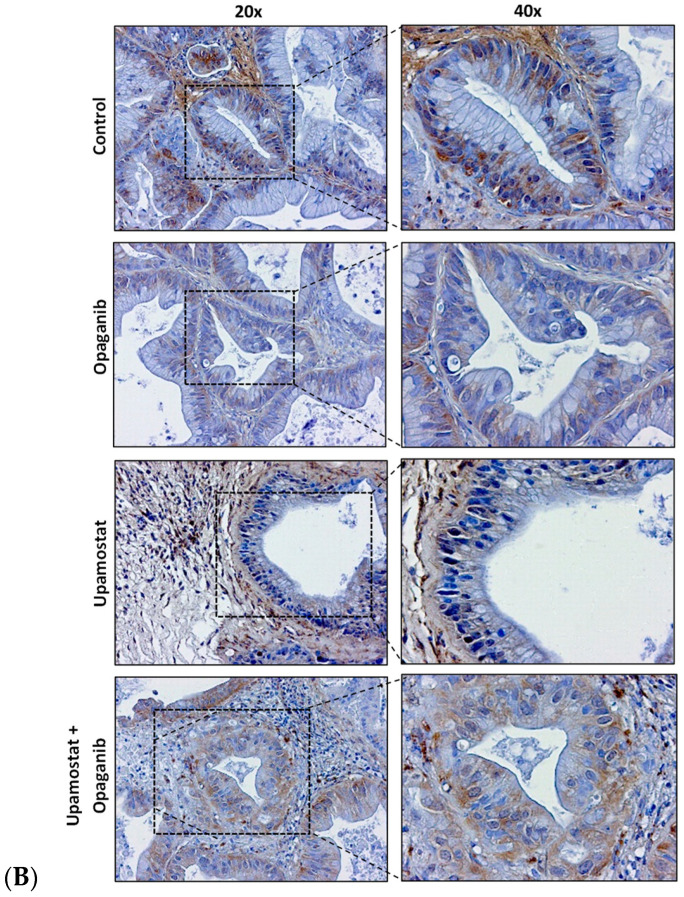
IHC staining of bile ducts shows decreases in drug target-expressing cells in CCA after treatment. Target: (**A**). Trypsin 1 (PRSS1), trypsin 3 (PRSS3), and putative trypsin 6 (PRSS3P2); (**B**). Sphingosine kinase 2 (SPHK2).

Immunohistochemical staining was performed to evaluate the expression of trypsin 1/3 and SPHK2 in CCA tissues. The staining confirmed the specificity of opaganib and upamostat for their primary targets, sphingosine kinase, and serine protease, respectively. The upamostat treatment group showed lower numbers of trypsin 1/3 (PRSS1/3) positive cells (Figure 3A), whereas significantly fewer SPHK2-expressing cells were observed in the opaganib treatment group (Figure 3B).

### 3.5. Opaganib and Upamostat Inhibit Proliferation and Induce Apoptosis in CCA Tumors

**Figure 4:**
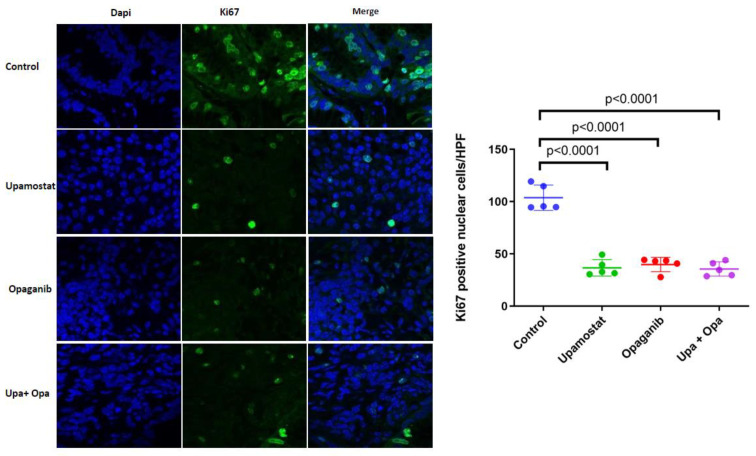
Upamostat, opaganib, and upamostat plus opaganib treatment groups showed significantly less Ki-67 staining, indicating reduced proliferation after treatment, *p* < 0.0001.

Using PAX165, which expresses high levels of SPHK2 and trypsin 1/2/3 (PRSS1/2/3), immunofluorescence staining was performed to investigate the expression of Ki-67 cell proliferation markers in the treated tissues. In the Ki-67 assay, the control group showed a significant level of Ki-67-positive cells, indicating a high level of cellular proliferation. Conversely, in the treatment groups, Ki-67-positive cells were significantly reduced (*p* < 0.0001, Figure 4). This suggests that serine protease inhibition and/or sphingosine kinase 2 inhibition of upamostat and opaganib inhibit(s) cellular proliferation, leading to a decrease in the number of actively dividing cells. 

**Figure 5:**
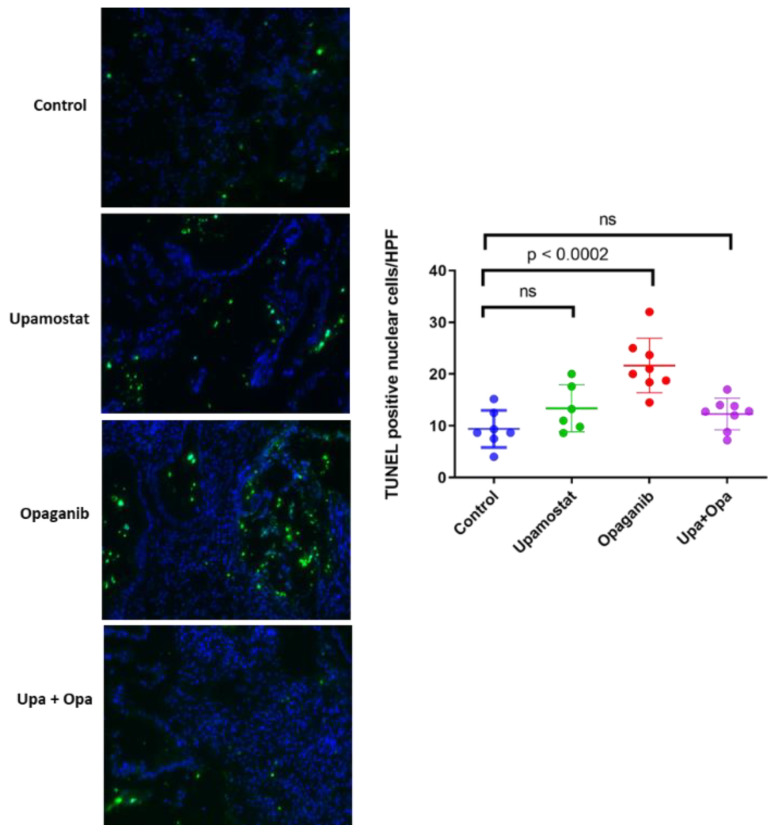
Significantly increased cellular apoptosis after opaganib treatment compared to all other treatment groups.

To measure the effect of upamostat and opaganib on cell apoptosis, we conducted a TUNEL assay. We found that opaganib induced a significantly higher number of TUNEL-positive cells as compared to the control group, with a *p* < 0.0002 (Figure 5).

### 3.6. Pharmacokinetic (PK) Analysis Confirmed That Upamostat Is Metabolized to WX-UK1

A PK study was conducted to evaluate the distribution and metabolism of opaganib and upamostat. This showed an accumulation of upamostat and WX-UK1 in the tumor as well as in liver tissue. WX-UK1 also accumulated in muscle tissue, suggesting that upamostat is metabolized to WX-UK1. This is consistent with WX-UK1 being the active metabolite of upamostat (Table S1).

### 3.7. Cell Viability and Migration in CCA Cell Lines

**Figure 6:**
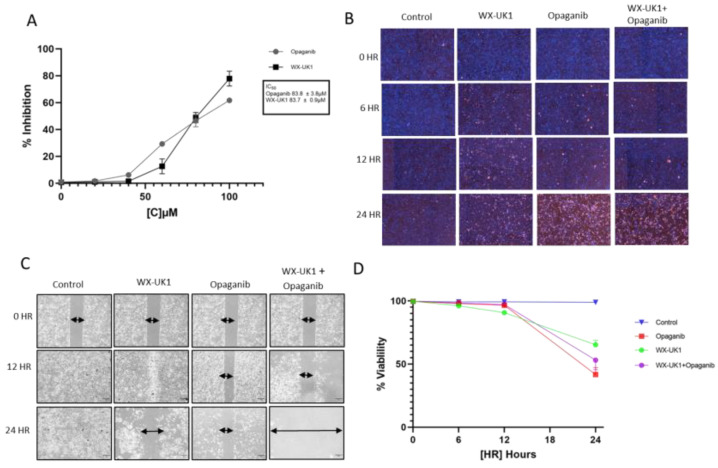
(**A**) IC50 of opaganib and the upamostat active metabolite, WX-UK1 in the HuCCT1 CCA cell line. (**B**,**D**) As indicated by an increase in the fluorescence of dead cells, all drug treatments decreased cell viability in CCA cell lines. (**C**) As indicated by a decrease in cells, WX-UK1 and opaganib in combination inhibit CCA cell migration (scale 300 µm).

The IC50 was assessed to determine the appropriate drug concentrations to test and analyze drug efficacy in cholangiocarcinoma cell lines. Inhibition analysis of opaganib and the upamostat active metabolite, WX-UK1, in the HuCCT1 CCA cell line, showed an IC50 of around 83 µM for both (Figure 6A).

Opaganib, WX-UK1 alone, and combination treatments were associated with decreased cell viability of CCA cell lines as indicated by increased fluorescence of dead cells (Figure 6B,D).

A migration assay was used to determine CCA cell proliferation after periods of 0, 12, and 24 h. HuCCT1 cells showed decreased migration when treated with a combination of WX-UK1 and opaganib (Figure 6C).

## 4. Discussion

CCA is a highly aggressive cancer with a poor prognosis, a high rate of tumor recurrence, and limited treatment options [31]. One approach to CCA treatment is to target specific highly expressed molecules that are critical for disease pathogenesis. The antitumor effects and safety of upamostat and its active metabolite WX-UK1 have been shown in multiple in vitro, preclinical, and human studies [8,32,33,34,35,36,37,38,39,40,41,42,43,44,45]. 

For this study of a CCA tumor PDX with the highest expression levels of our drug targets, we observed high levels of trypsin 3 (PRSS3) in the PAX165 tumor (Figure 1). Additionally, trypsin 3 was expressed at high levels in all the evaluated PDX tumors (Figure 3). This finding is particularly important and represents a novel observation in CCA tumors. Trypsin 3 expression is well established in the growth and metastasis of pancreatic tumors [46], but to the best of our knowledge, it has never been reported in CCA tumors. Serine proteases are known to be associated with cancer progression and metastasis [9]. Our group previously showed that WX-UK1, which is the active metabolite of upamostat (Mesupron), is a potent and specific inhibitor of five human proteases (trypsin-3, trypsin-2, trypsin-1, matriptase-1, and trypsin-6). Upamostat is the only known inhibitor of trypsin 3 that has reached clinical development [47]. 

Our group and others have previously demonstrated the overexpression of sphingosine kinase 2 (SPHK2) in CCA [22,23]. Using the first-in-class Sphk2 specific inhibitor (ABC294640, opaganib, Yeliva), we demonstrated in vitro inhibition of CCA cell proliferation [22,23]. Finally, Phase I studies of ABC294640 in solid tumors demonstrated the most significant response in CCA tumors [24]. Based on these preliminary data, we hypothesized that a combination of upamostat and opaganib may be therapeutically effective for reducing CCA solid tumor growth. In the present work, we selected a patient-derived CCA tumor based on the significant expression of the aforementioned targets and developed a mouse–human xenograft model of CCA. Herein, we show that upamostat and opaganib suppress CCA tumor growth in mice. Compared to the non-treated group, upamostat, and opaganib, each significantly reduced tumor growth. By combining both drugs, an even more significant tumor growth reduction was achieved. Of the two drugs, upamostat had the least effect on weight loss and both drugs in combination did not significantly cause weight reduction in mice. The significant reduction in tumor volume achieved with both drugs in combination likely stems from the divergent antitumor effects of each drug, which produces a more enhanced effect. Additionally, analysis of the Ki-67 and TUNEL assays reveal that both drugs, individually and in combination, significantly reduce proliferation, as well as possible involvement of opaganib in cellular apoptosis, respectively. The combination treatment did not seem to induce a significant increase in apoptotic cells; however, this could be because TUNEL detects only apoptotic cells. It is possible that most of the cells in the combination treatment group might have passed all the apoptotic stages detectable by TUNEL. Trypsin 1/3 and SPHK 2 expression were also evaluated in CCA tissues, and reduced numbers of the relevant target-positive cells were observed in the upamostat and opaganib groups, respectively. The reduction in staining could also be due to the drugs impeding the binding of the respective antibodies to the cells. This strongly suggests that upamostat and opaganib have high affinities for Trypsin 3 and Sphk2. A suggested approach for obtaining additional data for validation of treatment efficacy in the clinical trial setting could involve obtaining pre-and-post-paired treatment CCA tumor samples in patients to assess the key markers described in this paper. 

Furthermore, an in vitro study demonstrated that the HuCCT1 CCA cell line is sensitive to opaganib and upamostat. WX-UK1, the active metabolite of upamostat and opaganib, has an inhibitory effect on cell viability and migration when the cells are treated individually or in combination. Treatment with WX-UK1 and opaganib individually both resulted in cell growth regression. The combination of both drugs produced a more potent effect, which suggests that studies to understand the complex mechanistic interactions between the different treatment groups are necessary.

## 5. Conclusions

In summary, we demonstrate that simultaneously targeting serine protease trypsin 3 and sphingosine kinase (SPHK 2) with upamostat and opaganib results in a more enhanced inhibition of CCA tumor growth in mice. The in vivo tumor growth reduction is associated with the inhibition of tumor cell proliferation and the induction of apoptosis. Importantly, when used in combination, we did not observe significant weight loss in mice. These studies support the clinical trial evaluation of the efficacy of combination treatment with both upamostat and opaganib for CCA. 

## Figures and Tables

**Table 1:** Short tandem repeat (STR) analysis to compare allele repeats at specific loci in DNA between the F2 generation and the implanted tumors.

	F2 Gen		Implanted Tumor	
Marker	Allele No. 1	Allele No. 2	Allele No. 1	Allele No. 2
AMEL	X	X	X	X
D3S1358	14	15	14	15
D1S1656	15	16	15	16
D2S441	14	16	14	16
D10S1248	16	16	16	16
D13S317	8	8	8	8
Penta E	12	14	12	14
D16S539	12	13	13	13
D18S51	13	13	13	13
D2S1338	17	23	17	23
CSF1PO	11	12	11	12
Penta D	9	10	9	10
TH01	7	7	7	7
vWA	18	20	18	20
D21S11	29	31.2	29	31.2
D7S820	8	8	8	8
D5S818	12	12	12	12
TPOX	8	8	8	8
D8S1179	13	15	13	15
D12S391	17	18	17	18
D19S433	14	15	14	15
FGA	21	23	21	23
D22S1045	16	17	16	17

## Data Availability

The data presented in this study are available in the article.

## Supplementary Materials

The following supporting information can be downloaded at https://www.mdpi.com/article/10.3390/cancers16051050/s1, Figure S1: Pathology of the original tumor and first-generation mouse xenograft. A. H&E stain of the original tumor from the patient. B. H&E stain of the first-generation mouse xenograft.; Table S1: Pharmacokinetic (PK) analysis.

## Appendix A

Pharmacokinetic (PK) analysis experimental design.

Reagents and materials:

Upamostat, WX-UK-1, and opaganib were provided by RedHill Biopharma, Ltd. Taxol (internal standard) and formic acid (95%) were purchased from Sigma Aldrich (St. Louis, MO). LC-MS grade methanol (MeOH) was purchased from Fisher Scientific (Pittsburgh, PA). UltraPure water was filtered using the Milli-Q IQ-7000 water purifier (Millipore Sigma, Saint Louis, MO, USA). Control mouse plasma was purchased from Valley Biomedical (Winchester, VA, USA).

Instrumentation:

The LC-MS system consisted of a Waters Acquity H class ultra-performance liquid chromatography (UPLC) system, containing a quaternary solvent manager and sample manager-FTN coupled to a Xevo TQ-S mass spectrometer (Waters, Milford, MA, USA) equipped with an electrospray ionization (ESI) source. Data were acquired and analyzed by Waters MassLynx v4.1 software.

Chromatographic conditions:

The liquid chromatographic separation of upamostat, its metabolite UK-1, and opaganib were conducted using an Acquity UPLC BEH C18 analytical column (2.1 × 100 mm, 1.7 µm Waters, Milford, MA, USA) at 40 °C, eluted with a gradient mobile phase composed of water containing 0.1% formic acid (A) and MeOH containing 0.1% formic acid (B) with a constant flow rate of 0.4 mL/min and a total run time of 13 min. The elution was initiated at 55% B and B was linearly increased from 55–72% for 6.5 min, held at 100% B for 2.1 min, and returned to initial conditions for 4.4 min. The autosampler temperature was 4 °C and the sample injection volume was 2 µL.

Detection of upamostat, UK-1, opaganib, and taxol was accomplished using a mass spectrometer in positive ESI mode with a capillary voltage of 1.3 kV, source temp of 150 °C, desolvation temp of 500 °C, cone gas flow of 150 L/h, and desolvation gas flow of 1000 L/h, using multiple reaction monitoring (MRM) with a dwell time of 0.044 s. The cone voltages and collision energies were determined by MassLynx–IntelliStart v4.1 software, upamostat (cone 2 V and collision 26 eV), UK-1 (cone 4 V and collision 46 eV), opaganib (cone 2 V and collision 40 eV), and taxol (cone 38 V and collision 58 eV). The MRM precursor and product ions were monitored at *m*/*z* 630.42 > 398.17 for upamostat, 614.36 > 175.16 for UK-1, 381.20 > 189.08 for opaganib, and 854.43 > 105.09 for taxol.

Stock solution preparation:

The primary stock solutions of upamostat (1 mg/mL in MeOH), UK-1 (1 mg/mL in MeOH), opaganib (1 mg/mL in MeOH), and taxol (IS) (1 mg/mL in MeOH) were prepared in 4 mL of amber-silanized glass vials and stored at −20 °C. Working standards were prepared by diluting the stock solution with 1:1 MeOH:H_2_O in 1.7 mL microcentrifuge tubes and stored at −20 °C.

Mouse plasma samples:

Mouse plasma samples were extracted as follows: 50 µL of plasma sample was added to Orochem’s 2 mL, 0.2 µm protein crash plate (Chrom tech, Apple Valley, MN, USA) well containing IS (1662 ng/mL) in 300 µL of MeOH. The protein crash plate was shaken on a plate shaker for 10 min at 800 rpm. Next, the samples on the crash plate were filtered using a positive-pressure vacuum apparatus and collected in a deep 96-well plate. Plasma standard curves containing upamostat (1–1000 ng/mL), UK-1 (1–1000 ng/mL), opaganib (1–1000 ng/mL), and IS (1425 ng/mL) were prepared daily for quantitation.
