# Supplementary material for: Combined Antitumor Effect of the Serine Protease Urokinase Inhibitor Upamostat and the Sphingosine Kinase 2 Inhibitor Opaganib on Cholangiocarcinoma Patient-Derived Xenografts
Source: Cancers (Basel). 2024 Mar 5;16(5):1050. doi: 10.3390/cancers16051050 (PMC10930726; doi:10.3390/cancers16051050)
Supplement: Supplementary file 1 [file cancers-16-01050-s001.zip › cancers-2866776-supplementary.pdf]

H&E staining was performed to compare the characteristics of the original tumor (F0) and the first-generation mouse xenograft (F1). H&E staining revealed that both tumors were morphologically very similar (Figure S1).

A. 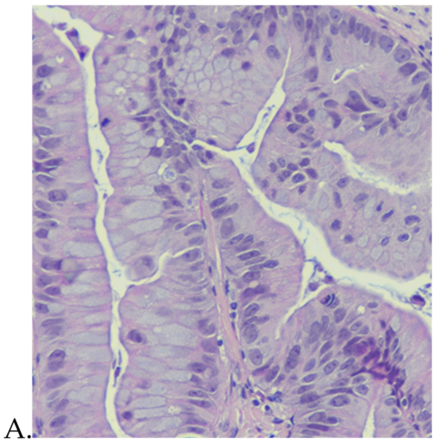 B. 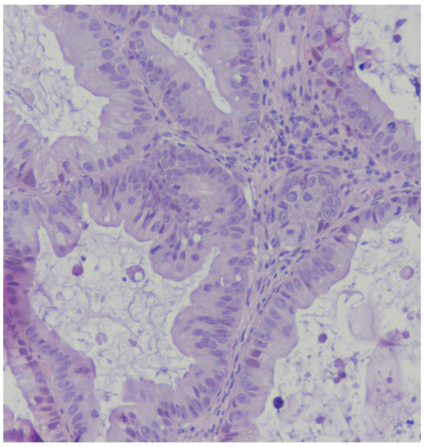

Figure S1. Pathology of the original tumor and first-generation mouse xenograft. **A.** H&E stain of the original tumor from the patient. **B.** H&E stain of the first-generation mouse xenograft

Table S1.: Pharmacokinetic (PK) analysis

Compound Administered	Animals /Dose mg/kg/adminstration	concentration(ng/g) Tumor Upamostat	WX-UK1	Opaganib	Liver Upamostat	WX-UK1	Opaganib	Muscle Upamostat	WX-UK1	Opaganib
Upamostat	70 mg/kg	61.0	66.3	0.0	8.8	83.9	0.0	<LOQ	102.4	0.0
Upamostat	70 mg/kg	55.8	68.3	0.0	9.3	69.5	0.0	<LOQ	26.6	<LOQ
Upamostat	70 mg/kg	10.0	68.6	<LOQ	14.9	56.9	<LOQ	<LOQ	8.6	<LOQ
Opaganib	50 mg/kg	<LOQ	<LOQ	4.8	<LOQ	0.0	4.8	0.0	0.0	<LOQ
Opaganib	50 mg/kg	<LOQ	<LOQ	<LOQ	0.0	0.0	6.7	0.0	0.0	<LOQ
Opaganib	50 mg/kg	0.0	0.0	4.6	0.0	0.0	<LOQ	0.0	0.0	<LOQ
Upamostat and Opaganib	70 mg/kg and 50 mg/kg	36.5	257.4	8.3	18.8	77.9	30.3	<LOQ	6.4	<LOQ
Upamostat and Opaganib	70 mg/kg and 50 mg/kg	9.5	130.3	<LOQ	<LOQ	30.5	<LOQ	0.0	6.4	<LOQ
Upamostat and Opaganib	70 mg/kg and 50 mg/kg	33.7	115.6	4.0	<LOQ	33.0	4.1	0.0	5.4	<LOQ
Control	Diluent	0.0	0.0	<LOQ	0.0	0.0	0.0	0.0	0.0	<LOQ
Control	Diluent	0.0	0.0	<LOQ	<LOQ	0.0	0.0	0.0	0.0	0.0
Control	Diluent	0.0	0.0	<LOQ	<LOQ	0.0	0.0	<LOQ	<LOQ	<LOQ
